# A Simple μ-PTV Setup to Estimate Single-Particle Charge of Triboelectrically Charged Particles

**DOI:** 10.3389/fchem.2019.00323

**Published:** 2019-05-07

**Authors:** Johann Landauer, Sandra Melina Tauwald, Petra Foerst

**Affiliations:** Chair of Process Systems Engineering, TUM School of Life Sciences Weihenstephan, Technical University of Munich, Freising, Germany

**Keywords:** triboelectric charging, triboelectric separation, singe-particle charge, charge distribution, charge measurement setup

## Abstract

Triboelectric separation is a useful phenomenon that can be used to separate fine powders. To design technical devices or evaluate the potential of powders to be triboelectrically separated, knowledge about the charge distribution on a single-particle level has to be obtained. To estimate the single-particle charge distribution in an application-oriented way, a simple μ-PTV system was developed. The designed setup consists of a dispersing and a charging unit using a Venturi nozzle and a tube, respectively, followed by a separation chamber. In the separation chamber, a homogenous electrical field leads to a deflection of the particles according to their individual charge. The trajectories of the particles are captured on single frames using microscope optics and a high-speed camera with a defined exposure time. The particles are illuminated using a laser beam combined with a cylindrical lens. The captured images enable simultaneous measurement of positively and negatively charged particles. The charge is calculated assuming a mean particle mass derived from the mean particle size. Initial experiments were carried out using starch of different botanical origins and protein powder. Single-component experiments with starch powders show very different charge distributions for positively and negatively charged particles, whereas protein powder shows bipolar charging. Different starch-protein mixtures show similar patterns for positive and negative charge distributions.

## 1. Introduction

Triboelectric charging occurs everywhere in nature from child rubbing a balloon on their hair to industrial powder handling. In particulate systems, triboelectric charging is predominately described as a problem, rather than an opportunity. Nevertheless, triboelectric charging is an exciting but so far not completely understood phenomenon. It has been investigated in many fields of research, such as contact electrification in dust devils (Farrell, 2004; Mareev and Dementyeva, 2017), in clouds after volcanic eruptions (Anderson et al., 1965; Mather and Harrison, 2006), in the formation of planets (Yair et al., 2008; Wang et al., 2017), and in almost every application dealing with fine and dry powders. Commonly, triboelectric charging is seen as a problem in industrial applications if particles are to be moved because charged particles tend to agglomerate and adhere on surfaces (Wong et al., 2015). Dry powder mixing (Ghori et al., 2014), pneumatic conveying (Bunchatheeravate et al., 2013; Korevaar et al., 2014; Grosshans and Papalexandris, 2016), and fluidised beds (Fotovat et al., 2017; Kolehmainen et al., 2017; Mehrani et al., 2017; Peltonen et al., 2018) are especially vulnerable steps in powder processing. In contrast, triboelectric charging of particles and surfaces is a desirable effect in electrophotography (Schein, 1999), nanogenerators (Wang, 2013; Jiang et al., 2018), and particle separation (Eichas and Schönert, 1992; Wu et al., 2013; Wang et al., 2015; Tabtabaei et al., 2016; Landauer and Foerst, 2018; Landauer et al., 2019).

In all cases, prediction of the triboelectric charging ability and characterization of charged particles is a necessary prerequisite for designing processes. Several approaches to measuring the charge of particles are known in literature. One simple charge-measuring setup is a Faraday cage or cup; when charged particles are put into a metal cup that is insulated on the outer wall, the electrical charge of the particles can drain and is measured. In the inductive one, particles flow through a conducting tube and the induced current can be measured. If both the charge of the Faraday cup and the mass of the particles is measured, the charge-to-mass ratio can be calculated. As it is a cumulative method, the measurement of bipolar charged particles is only possible with difficulty. For different applications, Faraday cups are designed to enable the measurement of fine powders or in a flow-through type (Matsuyama and Yamamoto, 1994; Watanabe et al., 2007). Faraday cups have been used in numerous studies to evaluate the charge-to-mass ratio (Zhao et al., 2002; Saini et al., 2008; Ducati et al., 2010; Ireland, 2010; Hussain et al., 2013; Pérez-Vaquero et al., 2016; Schella et al., 2017). Therefore, if particles are charged in a bipolar manner, the actual charge of a single particle is not determinable. Kelvin probe force microscopy is suitable for measuring surface charges. The charge present on a surface can be measured in high-resolution, but only in a small area (Nonnenmacher et al., 1991; Baytekin et al., 2012; Burgo et al., 2013; Mirkowska et al., 2014). However, Kelvin probe microscopy is an important method for illuminating the ongoing micro processes in triboelectric charging such as the *mosaic charged surfaces* (Baytekin et al., 2011), but only small surface sections can be characterized and the charging process can only happen in a standardized way. If the charge of a single particle must be evaluated, techniques inspired by characterization of particle movement or flow visualization can be used. In all cases, charged particles in motion are deflected in an electrical field depending on the ratio of mass to charge. In a pulsed electrical field, the frequency of the oscillating particle corresponds to the charge of the particle. The motion is measured by laser doppler velocimetry (Mazumder et al., 1991; Baron et al., 2011; Alois et al., 2017). In homogenous electrical fields, the charge-induced motion of particles can be visualized by consecutive images (Shin and Lee, 2002; Chull Ahn et al., 2004). The advantage of a particle-based charge measurement is the ability to calculate the charge distribution in a powder.

If particles are charged due to motion, a statistical contact probability between particles and the particle-wall has to be assumed, and the charge of all particles should be distributed. Furthermore, this distribution is also influenced by the colliding surface of the particles, potentially leading to eradication or single-sided increase of the particle charge after a bipolar charging step (Grosshans and Papalexandris, 2017). For large homogenous particles, charging and discharging is described by a combination of two different normal distributions (Haeberle et al., 2018). Conveyed particles also showed weight-normal distributions for chard silica and glassy carbon particles. An increase in gas velocity leads to a broadening of the charge distribution (Chull Ahn et al., 2004). Both cases show ideal particulate systems with probably very similar surface properties of individual particles. However, if triboelectric charging is to be used as a tailor-made tool to separate fine powders at a microscale size due to their triboelectric changing ability, a process-oriented measurement setup is needed to evaluate the possibility of powders for separation, as well as to design technical devices that incorporate the influences of different particle morphologies.

To gain a deeper insight into the charging mechanism and subsequent separation properties of powders of organic origin, a new measurement setup was designed. In order facilitate good comparability, the particle-charge measurement setup should have a similar design to earlier the described separation setups (Wang et al., 2014, 2015; Landauer and Foerst, 2018; Landauer et al., 2019). Furthermore, simultaneous measurement of a large number of positively and negatively charged particles is necessary. The designed setup should establish a basis for investigating the charge distribution of organic powders and their mixtures to evaluate the ability for separation. Starch granules from different botanical origin such as potato or corn, show dissimilar granular morphologies (Jane et al., 1994; Singh et al., 2003). Therefore, if particle morphology influences the triboelectric charging ability, the charge distribution of starch granules from different botanical origin might show different patterns. Consequently, binary mixtures of starch from different origins and proteins might show different charge distributions, as well as the influence of particle morphology upon triboelectric charging and subsequent separation.

## 2. Materials and Methods

### 2.1. Materials

Whey protein isolate with a protein content of 97.6 wt.% dry matter was purchased from Davisco Foods International, USA. Barley starch was purchased from Altia, Finland, with protein content below 0.5 wt.% and a starch content of 97 wt.% dry matter. Corn and potato starch were purchased with an analytical grade from Merck, Germany.

### 2.2. Methods

#### 2.2.1. Particle Charging

**Figure 1:**
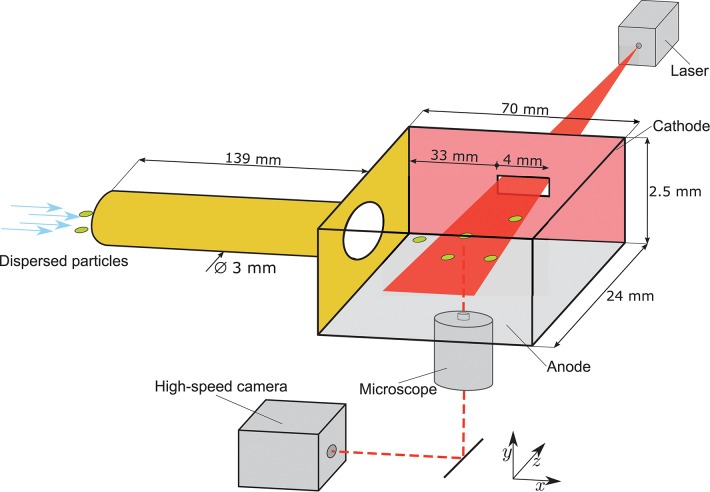
Experimental setup of the charging tube and the separation chamber and the micro particle tracking velocimetry (μ-PTV) setup to estimate the subsequent single-particle charge. The μ-PTV setup consists of a microscope with a high-speed camera and a sliced laser.

To measure the charge of single particles on the micrometer scale, a micro particle tracking velocimetry (μ-PTV) setup was developed. Figure 1 shows the experimental setup. The principle design is similar to the laboratory-scale separation unit shown in Landauer and Foerst (2018) and Landauer et al. (2019). Approximately 10 mg of powder is dispersed in a nitrogen stream using a Venturi nozzle and conveyed through a tube (Polymethylmethacrylat) with an inner diameter of 3 mm and a length of 139 mm. Such a small amount of powder does not strongly affect the homogeneity of the electrical field and the particle concentration in the measuring area is low. Hence, particle collisions that would be seen on the deviation of the path, can be excluded. The volumetric flow rate was adjusted to $\dot{V}$ = 0.8 m^3^ h^−1^. For all investigated powders, the charging conditions and the pretreatment were equal and thus differences in the charge distribution due to the setup and environmental conditions can be excluded.

#### 2.2.2. Particle Separation and μ-PTV

In the separation chamber, a homogeneous electrical field is applied perpendicular to the particle flow direction. To track the particle motion within the electrical field, a μ-PTV system is applied in the separation chamber. Therefore, the flow conditions in the separation chamber are adjusted to laminar flow (Re = 1,013). A He-Ne laser beam (Head 633-5P, Linos, Germany) with a diameter of 0.8 mm and a wavelength of λ = 632.8nm diverged by a cylindrical lens to a light sheet enters the separation chamber through a small slot in one electrode, which is in the same plane as the center of the tube. In order to track the particles, a high-speed camera (Phantom Miro 310, Vision Research, USA) with a frame rate of 3.200 Hz and an exposure time of 40 μs is used in combination with an inverse microscope (DM IRB, Leica Microsystems, Germany) at a magnification of 25x. The electrical field strength was set to 227 kV m^−1^. The middle region of the separation chamber was chosen as measuring area for the particles in order to avoid a non-homogenous electrical field. The relative permittivity of the used particles and gas are in the same range. Therefore, no influence of dielectrophoretic forces can be assumed. The resulting images show the trajectories of charged particles in the electrical field of the separation chamber.

**Figure 2:**
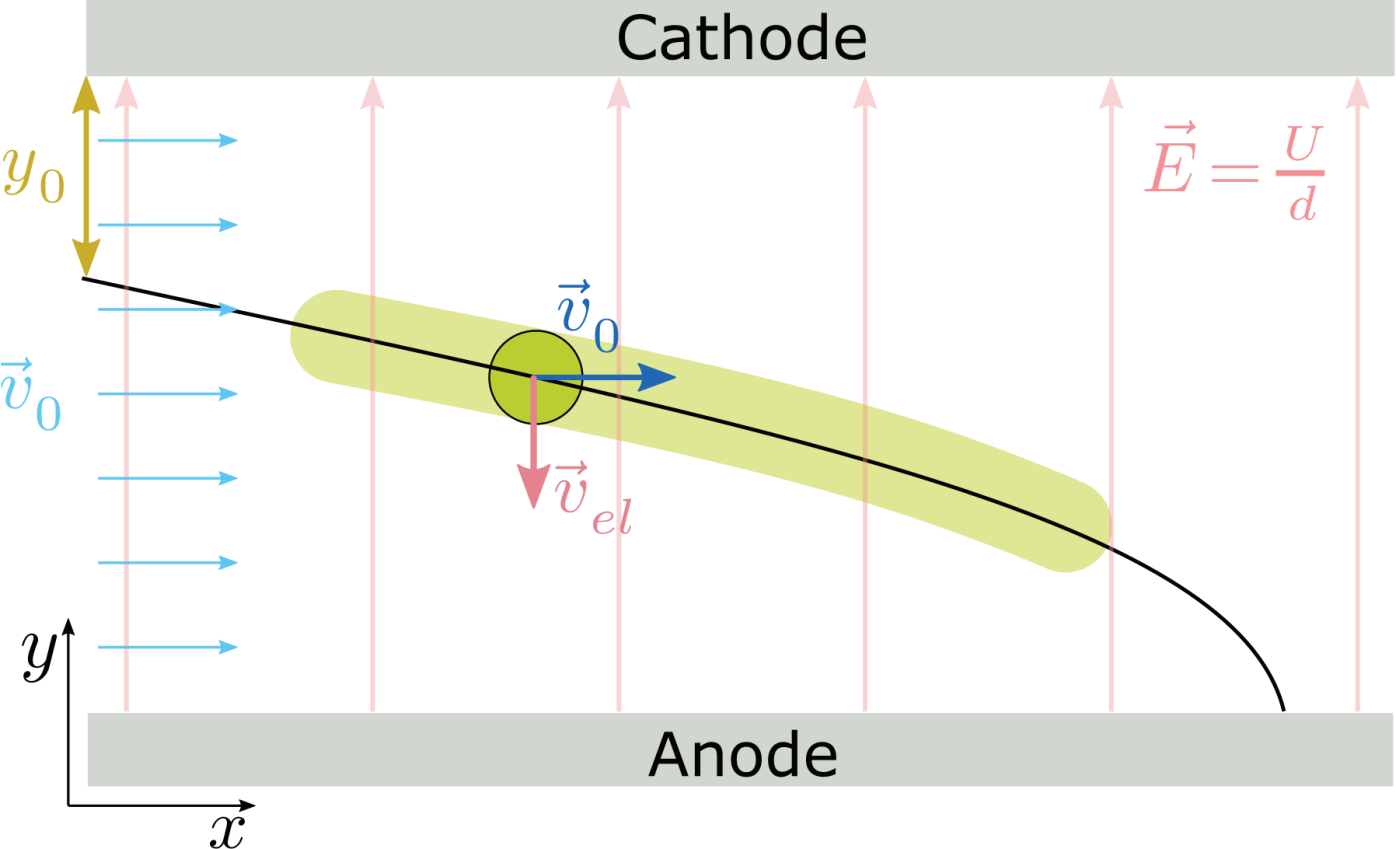
Scheme representing the evaluation from the images received in the μ-PTV setup including a charged particle (green) and the calculated trajectory of a single particle according Equation (3) with the recorded trajectory (transparent green) due to the adjusted exposure time. The black line indicates the resulting trajectory of a charged particle in the homogeneous electrical field $\vec{E}$ at a constant gas velocity $\vec{v_{0}}$. Velocity vectors attaching on the center of a particle indicate the motion in two spatial directions.

Figure 2 shows a schematic representation of the images received in the μ-PTV setup. The transparent green path was recorded with the camera. Within the homogeneous electrical field between the cathode and the anode, charged particles have a constant velocity $\vec{v_{0}}$ along the abscissa and an accelerated motion $\vec{v_{el}}$ along the ordinate. The resulting change of site $\frac{\partial \vec{r}}{\partial t}$ (1) yields a parabolic trajectory $\vec{r\left(t\right)}$ (3). The acceleration $\vec{a_{el}}$ of a particle of mass *m* carrying the charge *q* is induced by the electrical field strength $\vec{E}$ as the ratio of the voltage *U* and the distance of the electrodes *d*_*p*_ (2).

(1) $$\begin{array}{rc} \frac{\partial \vec{r}}{\partial t} & =\left(\begin{array}{c} \frac{\partial x}{\partial t}\\ \frac{\partial y}{\partial t} \end{array}\right)=\left(\begin{array}{c} v_{0}\\ \frac{\partial v_{el}}{\partial t} \end{array}\right)=\left(\begin{array}{c} v_{0}\\ a_{el}t \end{array}\right) \end{array}$$

(2) $$\begin{array}{rc} \vec{a_{el}} & =\frac{Uq}{\vec{d_{p}}\bar{m}} \end{array}$$

(3) $$\begin{array}{rc} \vec{r}\left(t\right) & =\left(\begin{array}{c} v_{0}t\\ \frac{U}{2\bar{m}d_{p}}qt^{2}+y_{0} \end{array}\right) \end{array}$$

To calculate the charge of each particle image processing of the images showing the path lines is used. First, to reduce *salt and pepper noise*, a linear median filter is used; Gaussian smoothing with σ = 1.5 is then employed to reduce further noise. Subsequently, the images are binarised using *adaptive thresholding*. These images are transformed into a skeleton and the resulting path lines are fitted to Equation (3) using the method of least squares (Figure 2, black line). The mass of one particle is calculated using the mean particle size and density. On raw images, no particles with a clearly detectable contour were visible, because only path lines were recorded. This contour is sharp. Therefore, it was impossible to calculate particle size based on the recorded path lines. Image processing is automated using MATLAB (MathWork, USA) in order to analyse high numbers of particles. Error bars of the charge distributions due to the variability of the particle size distribution were calculated according the standard derivation of the particle size distribution.

#### 2.2.3. Flow Simulation

In order to verify the assumption that turbulent flow dominates in the charging tube and laminar flow in the separation chamber, a CFD study using ANSYS Fluent, (ANSYS, Inc., USA) was carried out. In the CFD study the half of the symmetric geometry was simulated using a standard k-ϵ model with a standard wall function.

#### 2.2.4. Particle Characterization

To measure the particle size distribution of the powders, a laser diffraction system HELOS (Sympatec, Germany) with the RHODOS dispersing unit is used. True density is evaluated using a gas pycnometer (Accupyc 1330, Micromeritics Instrument Corp., USA). Scanning electron microscope (SEM) images were created using a JEOL JSM-IT100 (Japan) with a secondary electron detector at an acceleration voltage of 5 kV.

## 3. Results

### 3.1. Velocity and Turbulence Profile

**Figure 3:**
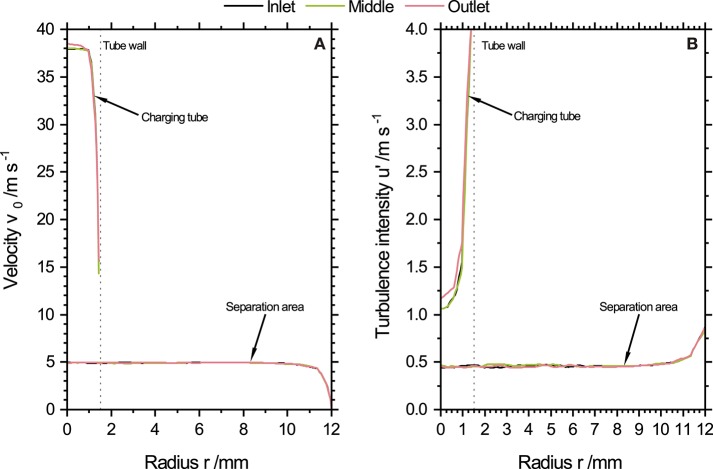
Velocity **(A)** and turbulence intensity **(B)** profile of the flow in the charging tube and the separation chamber. In the charging tube, high velocities and turbulence intensities were detected. In the separation/measuring chamber, moderate velocities and turbulence intensities were found. In the tube, a slight difference in the flow profiles between start (after six times the tube diameter after the beginning of the tube) and end (before six times the tube diameter of the end of the tube) is visible. In the measuring zone (positions are set due to the position of the gap for the laser (Figure 1), no differences were found.

Figure 3A shows the velocity profile and Figure 3 the turbulence intensity **B** $u^{\prime }=\sqrt{\frac{2}{3}k}$ calculated by the turbulence kinetic energy *k*. High turbulence intensities *u*′ indicate high Reynolds numbers, so direct effects of viscosity are negligibly small (Pope, 2000). The velocity profile in the charging tube shows slight differences between *inlet* and *outlet* of the tube, which is recorded six times the diameter after/before the inlet/outlet of the charging tube. In the center of the tube a gas velocity of 38 m s^−1^ is determined and high turbulence intensity is calculated. In contrast, the gas velocity in the separation chamber and especially at the inlet, middle, and outlet is 5 m s^−1^. This substantiates the assumption that there is a homogenous gas velocity [see Figure 2 and Equation (1)]. Drag force perpendicular to the flow direction could be excluded according to a Stokes number estimation.

### 3.2. Particle Characterization

**Figure 4:**
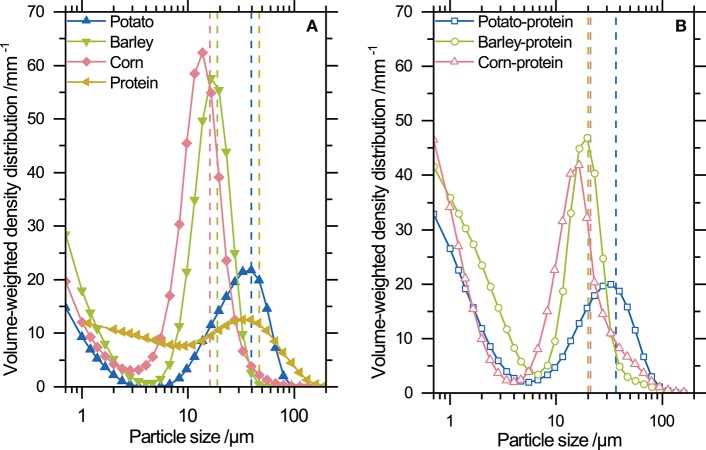
**(A)** Particle size distributions of the tested powders: potato, barley, and corn starch and whey protein. Each mean particle size *x*_50,3_ is indicated by dashed lines. **(B)** Particle size distribution of starch-protein mixtures containing 15 wt.% whey protein and potato, barley, and corn starch, respectively. Mean particle sizes are indicated by dashed lines.

Figure 4A shows the particle size distribution of the fine powders. Particle sizes of barley and corn starch are narrowly distributed between 4 and 40 μm. In contrast, potato starch and whey protein show a broader particle size distribution. Figure 4B shows the particle size distribution of starch-protein mixtures containing 15 wt.% protein. Table 1 shows a summary of the mean particle sizes, which were used to calculate the mean mass and the standard deviation of each particle size distribution. All distributions show a narrow size range. Table 1 summarizes the mean particle size and the standard deviation of the particle size distribution indicating the width of the distribution. Based on the standard deviation of the particle size distribution, the variation of the mean mass used for the estimation of the particle charge is approximately 4 times the mean. The mean mass $\bar{m}$ of an individual particle is calculated from the mean particle size *x*_50, 3_ and the true density under the assumption of spherical particles. The mean mass $\bar{m}$ of the individual particles is necessary to fit the recorded trajectories with Equation (3).

**Table 1:** Summary of the mean particle size and the related standard deviation of the particle size distribution.

	**Mean particle**	**Standard**
	**size /μm**	**deviation /μm**
Potato	39.5	10.7
Barley	18.9	6.5
Corn	15.9	6.2
Protein	46.8	16.8
Potato-Protein	36.6	15.7
Barley-Protein	20.4	11.0
Corn-Protein	21.1	11.2

### 3.3. Charge Distribution of Raw Powder

**Figure 5:**
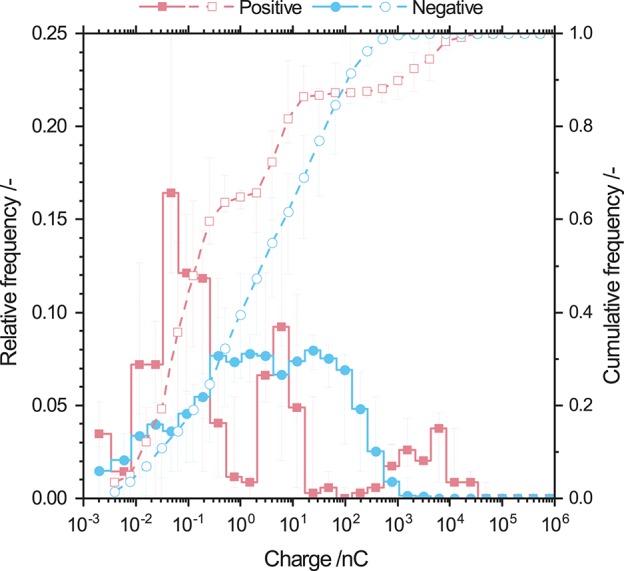
Relative and cumulative frequency distribution of positively and negatively charged barley starch particles. Positively charged particles show a trimodal distribution whereas negatively charged particles show a broad monomodal charge distribution. The charge of each particle was accumulated using a geometric series of basis 2. Error bars indicating the variation of charge due to the particle size distribution.

Figure 5 shows the charge distribution of both positively and negatively charged barley starch particles. Positively charged particles show a trimodal charge distribution with peaks from 10^−2^ to 5 × 10^−1^ nC, 2 to 2 × 10^1^ nC, and 2 × 10^2^ to 3 × 10^4^ nC. Positively charged barley starch particles show a monomodal charge distribution with a very wide range of charges from 10^−3^ to 10^3^ nC. The maximum of the relative frequency is located at 4 nC. The charge range of the negatively charged particles is smaller than that for positive ones.

Error bars indicate the variance of particle charge due to the variance in the particle size distribution (cf. Table 1). In order to improve readability of the figure in all further charge distributions show no error bars. For all investigated powders the charge distributions including error bars are shown in Figure 1S to point out that the charge distributions have a variance.

**Figure 6:**
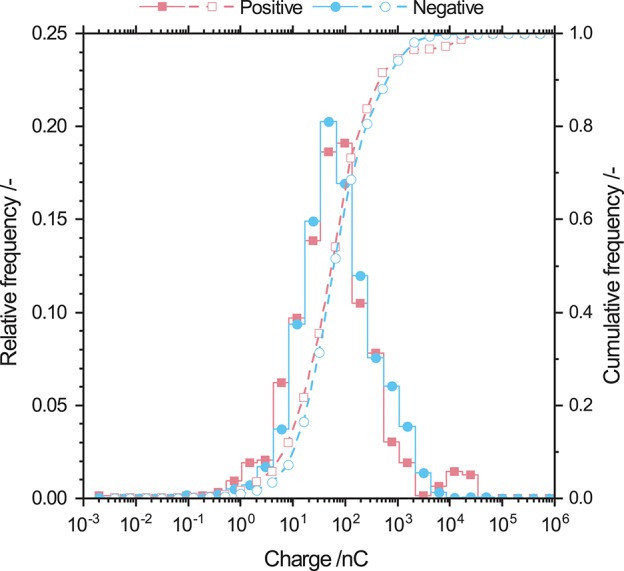
Relative and cumulative frequency distribution of positively and negatively charged whey protein particles. Such particles show the same monomodal charge distribution with a mean charge of 50 nC.

Different findings were obtained by analysing the charge distribution of protein powder shown in Figure 6. Positively and negatively charged particles show the same charge distribution with a mean charge of 50 nC. Furthermore, the span of the charge distribution has a narrow size range from 10^−1^ to 10^4^ nC.

**Figure 7:**
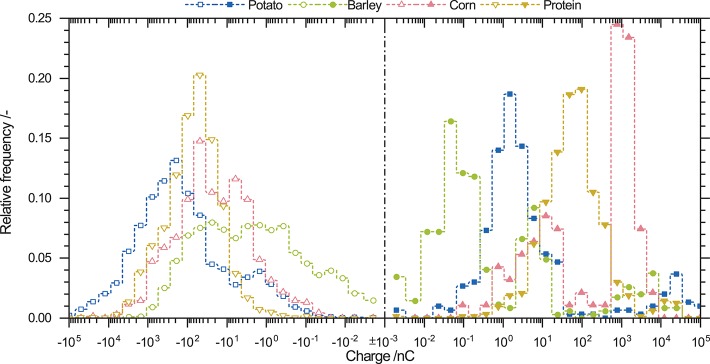
Charge distribution of potato, barley, and corn starch, as well as whey protein powder. After triboelectric charging negatively (open symbols) and positively (closed symbols) charged particles were found. Negatively charged particles show similar monomodal charge distributions, whereas positively charged particles show multimodal distributions.

Figure 7 shows a compilation of the charge distributions for all used raw powders. Negatively charged particles show a broad overlap and the monomodal distributions have the same shape. Potato starch particles have the highest charge and only a small number of particles possess a low negative charge (Table 2). Corn starch and protein particles have similar distributions for negatively charged particles. Barley starch particles show the smallest negative charge. By contrast, positively charged particles show completely different charge distributions. Particles of different origins do not overlap in a wide charge range. Besides barley starch, potato and corn starch also have trimodal and bimodal charge distributions, respectively. The highest positive charge is observed for potato starch followed by corn starch, barley starch, and protein. Corn, barley starch, and protein are on the same order of magnitude (Table 2). Thus, positively charged particles show a broad charge range depending on the used powder, whereas for negatively charged particles, a congruence in the charge distribution of all investigated powders can be observed.

**Table 2:** Summary of the first moment *M*_1,0_ of each charge distribution and the difference of the first moments Δ*M*_1,0_, indicating the net charge and the charge difference between positively and negatively charged particles, respectively. Furthermore, the numbers of particles evaluated to calculate the positive and negative charge distributions are displayed.

	***M*_1,0_** /**nC**	***ΔM*_1,0_** /**nC**	**Particle number /-**	**Total particle number/-**
Potato	4.61 × 10^4^	4.33 × 10^4^	300	3,874
	−2.86 × 10^3^		3,574	
Barley	6.69 × 10^2^	6.23 × 10^2^	347	2,430
	−4.51 × 10^1^		2,083	
Corn	9.19 × 10^2^	7.16 × 10^2^	94	792
	−2.03 × 10^2^		698	
Protein	6.68 × 10^2^	3.47 × 10^2^	628	2,957
	−3.21 × 10^2^		2,329	
Potato-Protein	2.65 × 10^3^	2.12 × 10^3^	94	792
	−5.34 × 10^2^		698	
Barley-Protein	3.72 × 10^3^	3.46 × 10^3^	300	3,181
	−2.62 × 10^2^		2,881	
Corn-Protein	7.04 × 10^4^	6.67 × 10^4^	626	4,381
	−3.67 × 10^3^		3,755	

Table 2 summarizes the means of each charge distribution, the difference of the means, and number of the measured particles. For raw powders, the mean of charge for positively and negatively charged particles are on almost the same order of magnitude. Differences in the mean are positive for all raw materials, indicating that positively charged particles carry a higher charge. Furthermore, this observation is underlined by comparing the numbers of simultaneously measured positively and negatively charged particles. Negatively charged particles possess an average percentage of the measured particle number of (87 ± 4) % and are thus attracted to the anode. Consequently, positively charged particles have an amount of (13 ± 4) %. Until now, the charge distributions of the four different raw powders exhibited similar findings for positively and negatively charged particles. The negatively charged particles showed a uniform monomodal charge distribution with broad overlaps, whereas positive charge was distributed unevenly over a wide range. The difference in the mean charge is always positive and the proportions of positively and negatively charged particles is uniform for all materials.

### 3.4. Charge Distribution of Powder Mixtures

**Figure 8:**
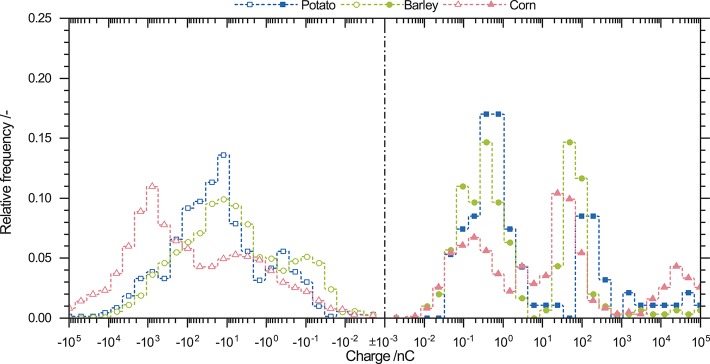
Relative frequency densities of positively and negatively charged particles originating from starch-protein mixtures containing 15 wt.% protein and potato, barley, and corn starch. Negatively charged particles have a wide monomodal charge distribution, whereas positively charged particles show a trimodal distribution.

Figure 8 shows the charge distribution of starch-protein mixtures containing 15 wt.% protein and starch from different origins. Negatively charged particles have monomodal distributions. The corn-protein mixture charge distribution has the highest negative mean charge; by contrast, barley-protein mixture has the lowest. These findings are differ from those for the mean charges of raw powders. Potato starch has the highest negative charge (Table 2). Charging of binary powders leads to an increase of negative charge, as indicated by an increase in the mean charge. As expected from the raw powder, barley starch has the highest number of weakly charged particles. Highly charged particles from raw potato starch (between −10^2^ and −10^4^ nC) are not visible in charging experiments using the starch-protein mixture. Corn starch-protein mixtures show a decrease in the relative frequency of particles with a charge between to −10^2^ nC and an increase in those with a charge between −10^2^ and −10^4^ nC.

Positively charged particles in Figure 8 exhibit different shapes of charge distributions compared to negatively charged ones. Starch-protein mixtures prepared with potato and barley starch had bimodal charge distributions with peaks approximately at the same positive charges of 1 *and* 10^2^ nC. By contrast, corn starch-protein mixtures have a trimodal charge distribution. Two peaks in potato and barley starch-protein mixtures are at approximately the same charge. The third peak is located at 2 × 10^4^ nC. The positive net charge for starch-protein mixtures increases from potato to barley to corn starch. The new peak at 10^2^ nC occurs where raw protein powder has its peak (Figure 7). This may indicate that protein as well as starch are positively charged.

Comparison of the charge distributions of binary mixtures of starch and protein an raw powders show that negatively and positively charged particles behave similarly. Negatively charged particles in both cases have monomodal and widely overlapping charge distributions. Positively charged particles form multimodal distributions. However, powder mixtures show uniform modes for all different starch particles, which may indicate the influence of protein particles on the amount of charge exchange onto positively charged particles.

## 4. Discussion

The guiding principle of the triboelectric effect is that if two surfaces come into contact and are separated subsequently, opposite charges remains on each surface. Thus, bipolar charging occurs. To examine triboelectric surface charging of fine powders with mean particle sizes between 20 and 40 µm (Figure 4), a novel experimental setup was established. This setup enables simultaneous measurement of the charges of positively and negatively charged particles; hence, a charge distribution for both positively and negatively charged particles can be calculated. However, some assumptions were made to calculate the charge of each particle. In order to calculate the mass of each particle, the mean of the volume-size distribution was used, which might lead to either an overestimation or underestimation of the particle's net charge. To remedy this inaccuracy, further work on optical resolution and illumination must be done. By improving the experimental setup to subsequently estimate the particle size and charge of each single particle, the direct correlation between size and charge can be evaluated. This might lead to deeper insights into the dependency of charge and particle size.

**Figure 9:**
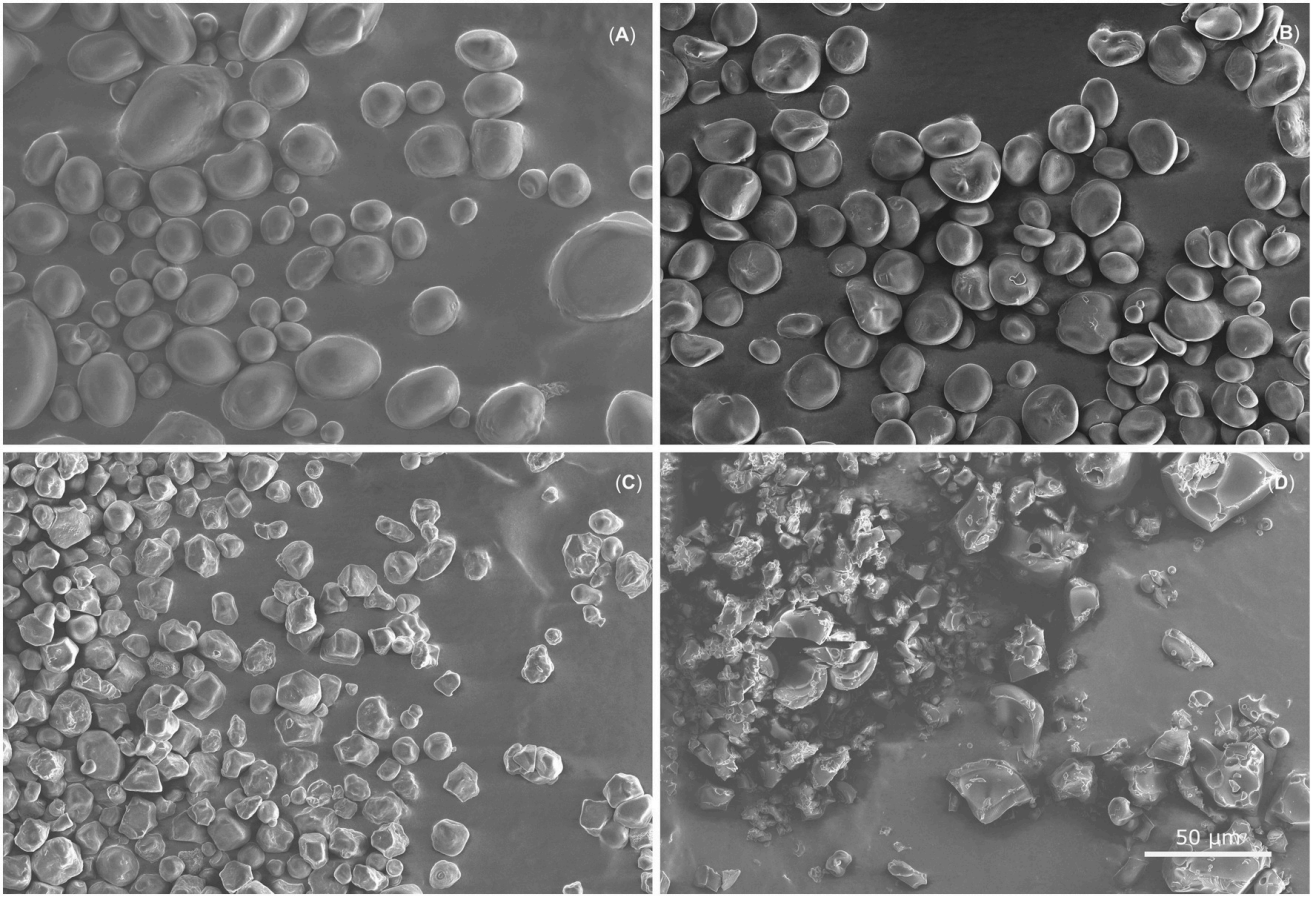
Scanning electron microscope images show raw powders of potato starch **(A)**, barley starch **(B)**, corn starch **(C)**, and protein **(D)**. Different particle morphologies between the different starch powders are clearly visible. Potato starch particles are both round and elliptical with a very smooth surface, whereas barley starch consists of round and lenticular particles with fine cracks on the smooth surfaces. Corn starch forms polyhedral particles with rough surfaces. Protein shows no principal particle morphology and different surface roughnesses are visible.

Experiments show completely different distribution patterns for positively and negatively charged particles. Negatively charged particles have monomodal charge distributions whereas positive ones show multimodal distributions. All pure-starch powders showed different charge distributions for positively and negatively charged particles. The empirical findings of monomodal and multimodal charge distributions are not deducible from previous studies. Thus, the setup allows to show another mosaic stone of triboelectric charging. In order to gain a deeper understanding of this pattern further studies are needed. It is well-known that, for bimodal powders, smaller particles become predominantly negatively and larger particles positively charged if both are present and particle-wall interaction is excluded (Lacks and Levandovsky, 2007). A decrease in mean particle size might accordingly lead to an increase in negative charge, but potato starch particles with the highest mean particle size show the highest negative charge (Table 2). However, it cannot be ruled out that other particle characteristics, such as morphology and crystallinity may influence triboelectric charging. SEM images (Figure 9) show different morphologies of the used pure-starch powders. It is well-known that starch from different botanical origins have different particle morphologies (Jane et al., 1994; Singh et al., 2003). The difference in morphology is influenced by the chemical structure of the monomers (amylose, amylopectin) forming the particles and their crystalline structure which molds the architecture and the surface properties of starch particles (Tang et al., 2006). This morphological difference might lead to different charge distributions. In comparison to starch particles, protein particles show bipolar charging (Figure 6); here, no clear difference in morphology is visible, as it is usual for powders performed by grinding agglomerates. Besides the morphological differences the between different powders, for every powder different particle sizes are present so an influence of the particle size could not be excluded.

For powder mixtures containing starch and protein, the same findings were made (Figure 8). The negative charge distribution is monomodal whereas the positive is trimodal. However, comparing powder mixtures with pure powders, same peaks are only visible for barley. Hence, binary powder mixtures lead to uniform charge distributions. The partially uniform distributions for both negatively and positively charged particles might suggest independence from the initial particle morphology, as shown for pure powders. Thus, the differences in the triboelectric charging abilities between starch and protein are more pronounced than the inhomogeneities of starch particles different botanical origins (Figure 9). However, an increase in the charge of the binary mixtures is not observed for all powder mixtures as should be expected (Table 2) (Forward et al., 2009). It is likely that the triboelectric charging properties of pure powders and binary mixtures are more complex and comprise different factors such as the chemical composition or morphology of the surfaces.

The experimental conditions for all carried out experiments were identical. All investigated powders had the same pretreatment, charging (turbulence intensity, flow rate, particle concentration), and environmental conditions. Thus, the influence of well-known influencing factors like humidity, particle interaction parameters, distinction between particle-particle and particle-wall interaction (Landauer et al., 2019), and reaching the saturation of charge on the particles were deliberately excluded in the proposed discussion. Since triboelectric charging is a surface phenomenon, it is assumed that the differences in morphology and composition of the surface influence the triboelectric charging and thus lead to differences in charge distributions. However, the setup enables to investigate these well-known influencing factors in further studies to gain a deeper insight in triboelectric charging.

## 5. Conclusion

In the present study, a novel measurement setup is presented to evaluate the charge of single particles in a size range between 1 and 100 μm. The setup based on a μ-PTV configuration enables each particle to be tracked and to have its associated charge calculated by the mean mass of the initial particle size distribution. The particles are dispersed in the gas phase for charge estimation; thus, this method can be adjusted to estimate the charge of all particles dispersed in the gas phase. However, uncertainties in the evaluation arise due to the unknown relationship between the recorded trajectory and the exact particle size or mass. To overcome the deficiency of this setup, the velocity in the measuring area has to be reduced but simultaneously the turbulence intensity, which ensures a high particle-particle interaction rate, should be kept constant. For all calculations, the mean particle mass assuming a homogeneous true density of the used powders was employed. Nevertheless, the novel measurement setup enables the charge distributions of triboelectrically charged powders to be calculated.

Charge distributions of large portions of all investigated triboelectrically charged powders showed the same pattern. Negatively charged particles have a monomodal distribution whereas positively charged ones have a multimodal one. These findings were the same for both raw powders and starch-protein mixtures. Comparing different raw starch powders, different triboelectric charging properties are visible. It is assumed that different particle morphologies may be the reason, because all further influence parameters were the same for performed experiments. Analysis of the starch-protein mixtures exhibits no such tendency of different particle morphologies. It is likely that particle-particle interaction of particles of different botanical origin mask the impact of particle morphologies and sizes. The developed setup to estimate the single particle charge can be a valuable tool to gain a deeper understanding of triboelectric charging. In further studies the setup can help to investigate several questions in triboelectric charging like influence of interaction rate, charging time or saturation of charge.

## Author Contributions

JL did the conception and design of the study and wrote the manuscript. ST did the experimental works. PF participated to the writing and supervised the work.

### Conflict of Interest Statement

The authors declare that the research was conducted in the absence of any commercial or financial relationships that could be construed as a potential conflict of interest.
