# Supplementary material for: A Simple μ-PTV Setup to Estimate Single-Particle Charge of Triboelectrically Charged Particles
Source: Front Chem. 2019 May 7;7:323. doi: 10.3389/fchem.2019.00323 (PMC6514107; doi:10.3389/fchem.2019.00323)
Supplement: Supplementary file 1 [file Data_Sheet_1.pdf]

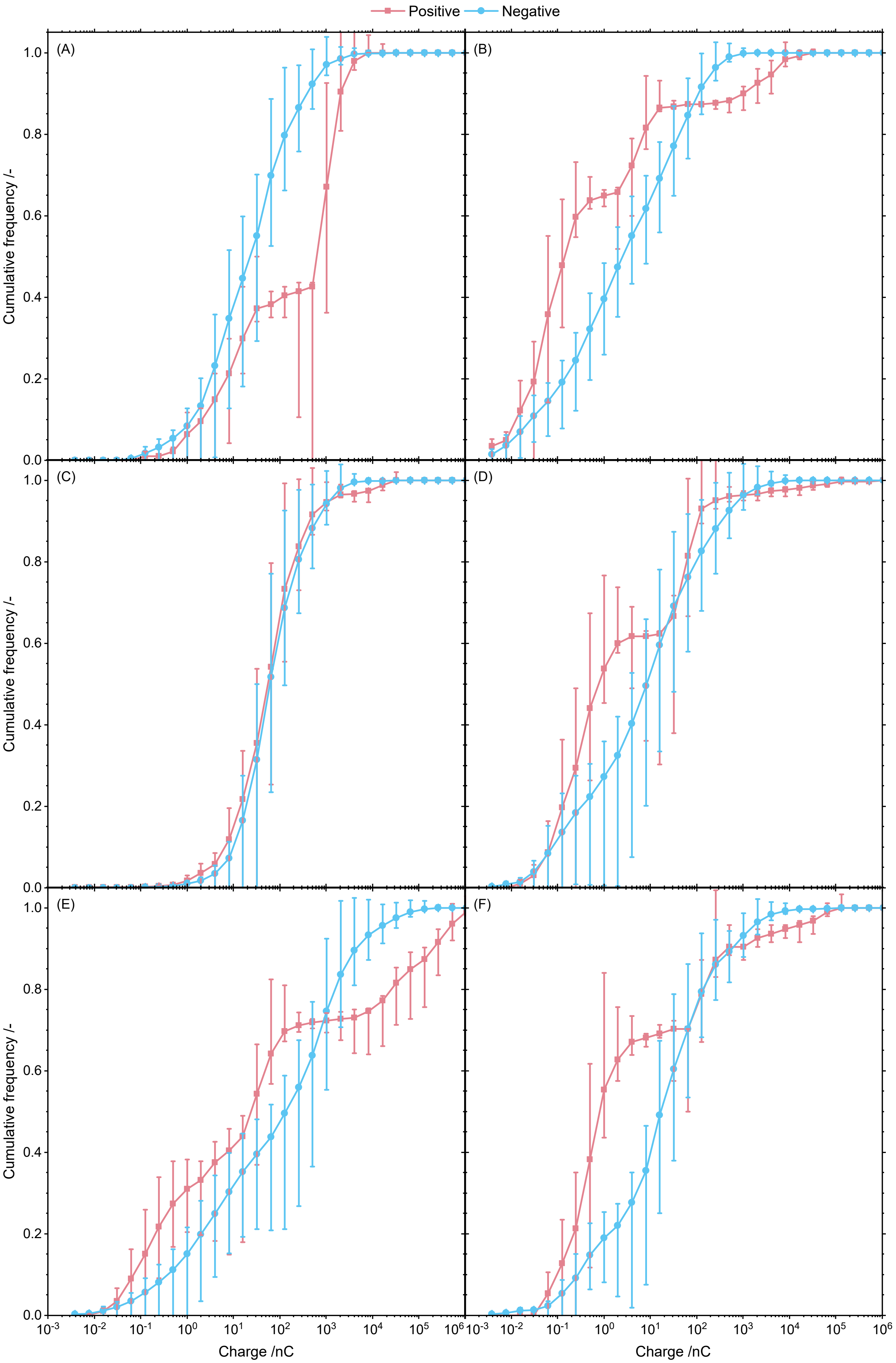

Figure 1S: Cumulative frequency of corn starch (A), potato starch (B), and whey protein (C) as well as starch-protein mixtures containing 15 wt.% protein and barley (D), corn (E), and potato starch (F). Error bars indicating the uncertainty in calculating the charge using data of a particle size distribution with a given standard derivation.
